# ceAF Ameliorates Diabetic Wound Healing by Alleviating Inflammation and Oxidative Stress via TLR4/NF-*κ*B and Nrf2 Pathways

**DOI:** 10.1155/2023/2422303

**Published:** 2023-04-05

**Authors:** Shiyan Li, Xiaofeng Ding, Xin Yan, Jin Qian, Qian Tan

**Affiliations:** ^1^Department of Burns and Plastic Surgery, Nanjing Drum Tower Hospital, the Affiliated Hospital of Nanjing University Medical School, NO. 321, Zhongshan Road, Nanjing, Jiangsu, China; ^2^Department of Burns and Plastic Surgery, Nanjing Drum Tower Hospital Clinical College of Traditional Chinese and Western Medicine, Nanjing University of Chinese Medicine, NO. 321, Zhongshan Road, Nanjing, Jiangsu, China; ^3^Anhui Hygeiancells BioMedical Co. Ltd., Huangshan, Anhui, China; ^4^Stem Cell Application Research Center, The Hangzhou Branch of Yangtze Delta Region Institute of Tsinghua University, Hangzhou, Zhejiang 310019, China; ^5^Department of Burns and Plastic Surgery, Anqing Shihua Hospital, Nanjing Drum Tower Hospital Group, Anqing 246002, China

## Abstract

**Background:**

With the rise in diabetes incidence, diabetic foot ulcers have become the most common clinically chronic refractory wounds. Persistent chronic inflammation is a typical feature of diabetic cutaneous wounds, and diabetic wound healing can be improved by alleviating inflammation and oxidative stress. Chick early amniotic fluids (ceAF) consist of native conglutinant substances with balanced amounts of growth factors, cytokines, and chemokines. However, whether ceAF modulates inflammation and oxidative stress and thus promotes diabetic wound healing remains unknown.

**Materials and Methods:**

RAW264.7 cells were categorized into four groups: negative control, LPS, LPS + ceAF, and ceAF. 10% of ceAF was selected to treat different groups of mice with a full-thickness skin defect wound. Then, RT-qPCR, western blot, immunofluorescence, and other assays were carried out to explore the effect of ceAF on wound healing and its molecular mechanism.

**Results:**

Topical administration of ceAF improved M2 macrophage polarization and inflammatory response in the wound tissues, thereby ameliorating delayed wound healing. Histological improvement could be observed in the grade of inflammation, collagen deposition, and neovascularization in wound edge tissues. ceAF also increased M2 macrophage-specific markers expression and exogenous ceAF suppressed LPS-induced cellular inflammatory response *in vitro* high glucose environment. Additionally, ceAF could activate TLR4/NF-*κ*B and Nrf2 signal transductions to promote M2 macrophage polarization *in vitro*.

**Conclusions:**

In summary, ceAF downregulates inflammatory response, regulates M2 macrophage transition via TLR4/NF-*κ*B and Nrf2 signaling pathways, and thus improves diabetic wound healing.

## 1. Introduction

As a kind of chronic wound, the diabetic wound has been vexing diabetic patients and clinicians [1]. Due to the complex microenvironment of diabetic wounds such as hypoxia, infection, ischemic condition, inflammation, and oxidative stress, diabetic ulcer is often prolonged and recurrent [2, 3]. Traditional debridement and dressing change therapy has limited effect on diabetic wounds [4]. The application of the flap is also dependent on the patient's own condition because occlusion of distal blood vessels in the limb is common in diabetics [5]. Therefore, how to treat diabetic ulcer efficiently and economically has become an urgent problem.

Oxidative stress and inflammation are closely related to wound healing in diabetes mellitus [6]. According to the activators, macrophages can be classified into classically-activated M1 or alternatively-activated M2 phenotypes [7]. During wound healing, M1 macrophages are responsible for the phagocytosis of necrotic tissue and cell debris, while M2 macrophages are involved in inhibiting inflammation and promoting tissue regeneration [8]. Diabetic wounds are often in a state of excessive inflammation. There is a large amount of M1 macrophages in wound tissue and the transformation of macrophages to M2 type is interrupted [9]. Notably, previous research has demonstrated that increasing the M2 phenotype could be a vital factor in diabetic wound repair [10, 11]. The combination of suppressing oxidative stress damage and promoting M2 macrophage polarization can be desirable for diabetic ulcer.

Transcription factors Nrf2 and NF-*κ*B are classical prototypical proinflammatory effectors that regulate cell proliferation, apoptosis, and differentiation [12]. TLR4 is an important upstream regulator of NF-*κ*B signal transduction [13]. Previous reports have shown that TLR4/NF-*κ*B signal path is associated with macrophage polarization [14, 15]. The induction of iNOS in macrophages was dependent on NF-*κ*B signal transduction [16]. miR-146a can promote M2 macrophage polarization through inhibiting TLR4/NF-*κ*B axis during diabetic wound healing [17]. Heme oxygenase 1(HO-1) is regulated by Nrf2 and plays an important immune-modulatory role in macrophages; the induction of HO-1 could switch these cells from the proinflammatory (M1) to anti-inflammatory (M2) phenotype. [18].

In many species, amniotic fluid is a nourishing, protective fluid that surrounds the embryo throughout the pregnancy [19]. It contains stem cells derived from embryos, and thus, is a favorable substance for wound healing [20]. ceAF is a compound that we extract from chick embryos that have been incubated for 6-8 days. In view of the previous studies mentioned that embryonic amniotic fluid contains stem cells and growth factors which were conducive to wound healing. We designed experiments on wound healing *in vivo* and *in vitro* to investigate the therapeutic effects and possible mechanisms of ceAF.

## 2. Materials and Methods

### 2.1. Antibodies and Reagents

APC CD206 antibody and FITC F4/80 antibody were procured from eBioscience. Arginase1 (Arg-1), *α*-SMA, and CD206 were purchased from CST. GAPDH, iNOS, TNF-*α*, IL-6, IL-1*β*, Nrf2, HO-1, NQO1, and CD31 were purchased from Abcam. TLR4, NF-*κ*B-p65, and p-IKB were purchased from ABclonal. The Trizol Reagent and SYBR green were purchased from Vazyme Biotech. STZ and glucose were purchased from Sigma-Aldrich.

### 2.2. Preparation of ceAF

Fertile chicken eggs were hatched at 38°C and 50% humidity. Between days 6 and 8 of hatching, ceAF was isolated from the eggs. After centrifugation (2500 × g, 20 min), a 0.22 *μ*m sterile filter (Millipore, USA) was used to filter the supernatant. The filtered specimens were aliquoted and kept at -80°C.

### 2.3. Cell Culture

RAW264.7 cells were supplied by the Chinese Academy of Sciences Cell Bank and then cultured in high-glucose DMEM medium containing 10% of FBS and 1% of double antibody at 37°C with 5% of CO_2_. The DMEM with 40 mM glucose was employed as high-glucose conditions. Various concentrations of ceAF were added to the medium for subsequent experiments.

### 2.4. Animals and Wound Procedure

The experimental protocols were approved by the Animal Care and Ethics Committee of Nanjing University. C57BL/6 mice (male, 8 weeks old) were obtained from the Model Animal Research Center of Nanjing University, and maintained in a specific pathogen-free environment with unlimited access to water and food. Eighteen mice in each group were injected intraperitoneally every day with 50 mg/kg STZ (in sodium citrate buffer) for five days in order to construct an STZ-induced diabetes model. The mice were given blood glucose measurements after three weeks, and those with blood glucose levels > 16.7 mM were classified as diabetes. To establish an excisional wound model, an 8 mm circular biopsy punch was performed on the back skin of mice following hair removal. After modeling, 10% ceAF was topically applied to the wound surface daily and the control mice were given equal volume PBS. The wound images were captured on days 0,3,5,7, and 11, and wound areas were measured with ImageJ software (National Institutes of Health). Wound tissue samples were collected on days 5 and 10 postinjury for subsequent experiments.

### 2.5. Histological and Immunofluorescent Staining

The wound margin tissues were fixed, dehydrated, embedded in paraffin, and sectioned at 5 *μ*m thickness. Masson's trichrome (MT), hematoxylin-eosin (H&E), and Sirius red staining were conducted according to standardized histological procedures. To assess macrophage polarization and angiogenesis, CD206, iNOS, CD31, and *α*-SMA monoclonal antibody (1 *μ*g/ml) staining was carried out at 4°C overnight. Then, a specific fluorescent secondary antibody was incubated, followed by DAPI staining.

RAW264.7 cells were sequentially rinsed with PBS, fixed in paraformaldehyde (4%), perforated with 0.1% Triton X-100, and blocked with BSA (3%). Then, the cells were incubated with corresponding primary and secondary antibodies according to the instructions. All photographs were taken using an Olympus FluoView FV3000 confocal microscope (Tokyo, Japan).

### 2.6. RNA Isolation and RT-qPCR

Cells and wound margin tissue were treated with Trizol Reagent to isolate total RNA by following the manufacturer's instructions. RT-qPCR was conducted with SYBR green dye using the StepOne RT-qPCR system (Applied Biosystems, USA). After normalization with GAPDH, the relative gene levels were determined using the 2^−*ΔΔ*CT^ method. The primer pairs are listed in Table 1.

### 2.7. Western Blot (WB) Analysis

Protein samples were isolated from lysed skin tissues and cells using RIPA lysis buffer (KeyGEN, China). BCA assay was performed to determine the total protein concentration after centrifugation. The protein specimens were separated through 10% SDS-PAGE gel and transferred onto the PVDF membrane (Millipore, USA). After blocking with 5% BSA, the membrane was incubated with the corresponding primary for overnight and secondary antibody for 1 h. The visualization of protein bands was conducted using an ECL substrate kit (Vazyme, China).

### 2.8. Flow Cytometry

To determine the polarization trend of RAW264.7 macrophages, the cells were preincubated with FITC-conjugated anti-mouse F4/80 antibody and APC-conjugated anti-mouse CD206 antibody at 4°C in the dark for 30 min. The cell phenotype was determined using a flow cytometer (FACSCanto II, BD, USA), and data analysis was conducted with FlowJo software.

### 2.9. Cell Viability Test

CCK-8 assays (Beyotime, China) were used to assess RAW264.7 cell viability. After 12 h of starvation, the cells were exposed to 0%, 1%, 5%, 10%, or 20% of ceAF and then incubated for 24 h. The cells were rinsed with PBS 3 times, and then covered with 200 *μ*L in a complete medium containing CCK-8 mixture (10 *μ*L) and incubated at 37°C. Absorbance was measured using a microplate reader at 450 nm.

### 2.10. Measurement of SOD, MPO, MDA, GSH-Px, and ROS Levels

RAW264.7 cells were stimulated with 100 ng/mL LPS for 48 h to induce cellular inflammation. Protein concentration was determined quantitatively with a BCA protein assay kit (Pierce Biotechnology, Rockford, Illinois, USA). SOD, MPO, MDA, GSH-Px, and ROS levels in cells were measured according to a previously described method using a relevant assay kit (Nanjing KeTeng Biotech Co. Ltd., Nanjing, China).

### 2.11. ELISA

RAW264.7 cells were exposed to 10% of ceAF for 48 h, and cellular supernatants were collected for testing. The secreted IL-6, IL-10, TGF-*β*1, and TNF-*α* were measured via ELISA kit according to the kit instruction (Elabscience, China).

### 2.12. Statistical Analysis

Experimental data were analyzed with GraphPad Prism v8.0 software and presented as mean ± SEM. Parametric tests were used for data that conform to the normal distribution (Shapiro–Wilk test). If the data were normally distributed, the statistical differences among multiple groups were compared with one-way ANOVA and Newman-Keuls post hoc test. The two-tailed Student's t test was utilized for the comparison of two groups if the data passed the normality test. The combined effects of two factors were analyzed with two-way ANOVA followed by Tukey's posttest. At least three independent assays were conducted, and *P* values of <0.05 were defined as statistically significant.

## 3. Results

### 3.1. ceAF Attenuates the Inflammation and Oxidative Stress of LPS-Stimulated RAW 264.7 Cells via TLR4/NF-*κ*B and Nrf2 Axis

**Figure 1:**
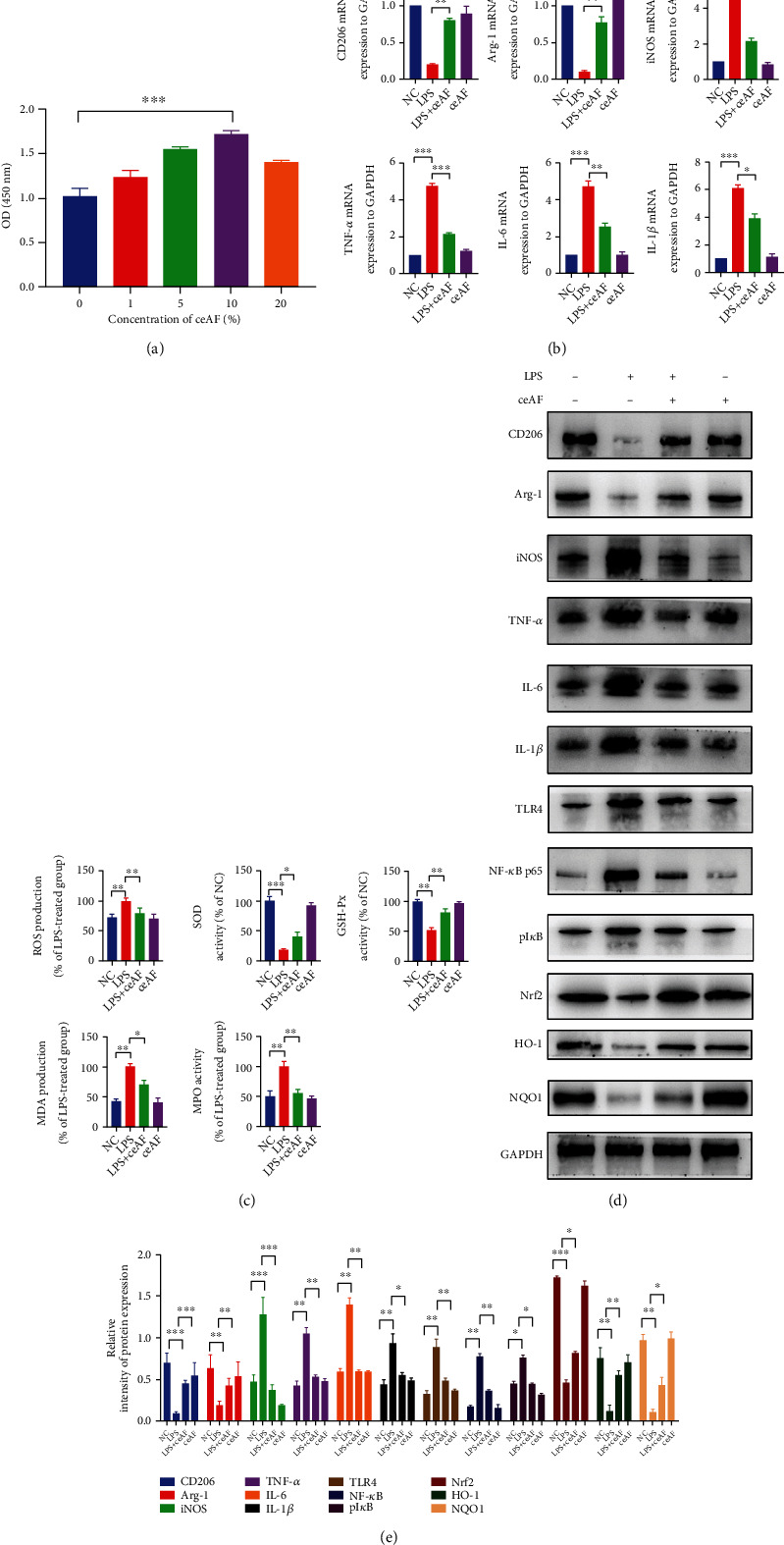
ceAF was able to suppress inflammation and oxidative stress via TLR4/NF-*κ*B and Nrf2 pathways in LPS-stimulated RAW 264.7 cells via TLR4/NF-*κ*B and Nrf2 pathways. (a) The viability of RAW 264.7 cells exposed to ceAF at various concentrations. (b) The expression levels of CD206, Arg-1, iNOS, IL-1*β*, IL-6, and TNF-*α* were assessed by RT-qPCR. (c) The levels of ROS SOD, GSH-Px, MDA, and MPO were measured. (d) WB results of the influence of ceAF on macrophage markers and TLR4/NF-*κ*B axis. CD206, Arg-1, iNOS, IL-1*β*, IL-6, TLR4, TNF-*α*, NF-*κ*B p65, pI*κ*B Nrf2, HO-1, and NQO1 were tested, and GAPDH served as a standard reference. (e) Bands from the WB in (d) were analyzed by densitometry. *n* = 3, Mean ± SEM. NC: negative control; ^∗^*P* < 0.05, ^∗∗^*P* < 0.01, ^∗∗∗^*P* < 0.001.

To explore the regulatory role of ceAF on LPS-induced cellular inflammation, we stimulated RAW264.7 with 100 ng/mL LPS for 48 h to induce cellular inflammation. Firstly, the proliferative capability of RAW264.7 cells was tested with different concentrations of ceAF. CCK8 results demonstrated that the proliferative capability of RAW264.7 increased with the progressive of ceAF concentration and reached the peak at 10% concentration (Figure 1(a)). Next, we validated macrophages and inflammatory markers at the transcriptional level. RT-qPCR analysis indicated that the mRNA levels of M2 macrophage markers (Arg-1 and CD206) were remarkably decreased in the LPS group and increased in the LPS+ceAF group (Figure 1(b)). While the mRNA levels of the M1 marker (iNOS) and inflammatory factors (IL-1*β*, IL-6, and TNF-*α*) were markedly decreased in the LPS+ceAF group (Figure 1(b)). LPS utilization significantly increased ROS, MDA, and MPO levels, while the levels of SOD and GSH-Px were significantly decreased, and these changes could be reversed by ceAF treatment (Figure 1(c)). Further, TLR4/NF-*κ*B pathway-associated proteins were significantly activated and Nrf2 pathway-associated proteins were markedly decreased in the LPS group. However, the trend was reversed in the LPS+ceAF group (Figures 1(d) and 1(e)).

### 3.2. ceAF Induces RAW264.7 to Polarize M2 Macrophages *In Vitro*

**Figure 2:**
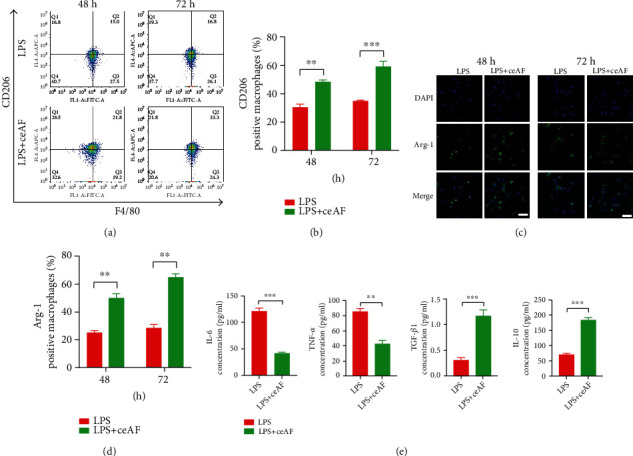
ceAF augments M2 macrophage polarization *in vitro*. (a) Flow cytometric analysis of M2 macrophage proportions after ceAF treatment in a time-dependent manner. (b) The quantified results of (a) are presented in a bar chart. (c) Immunofluorescence staining indicated that Arg-1^+^ cells were treated with LPS and LPS+ceAF at 48 and 72 h. Scale bar: 40 *μ*m. (d) The quantified results of (c) are presented in a bar chart. (e) ELISA detection results of IL-6, TNF-*α*, TGF-*β*1, and IL-10. *n* = 3, Mean ± SEM. ^∗∗^*P* < 0.01, ^∗∗∗^P < 0.001.

Next, flow cytometry was conducted to assess the polarization state of macrophages after exposure to LPS and ceAF. It was found that the proportion of M2 macrophages was greatly enhanced in the LPS+ceAF group than in the LPS group at 48 and 72 h (Figures 2(a) and 2(b)). Similar results of changes in macrophage polarization were also observed by immunofluorescent staining with reaching statistical significance (Figures 2(c) and 2(d)). We further detected the inflammatory-related cytokines in the cellular supernatant using ELISA. The secretion of TNF-*α* and IL-6 in the LPS+ceAF group was markedly attenuated compared to that in the LPS group (Figure 2(e)). On the contrary, TGF-*β*1 and IL-10 were dramatically increased as anti-inflammatory factors in the LPS+ceAF group (Figure 2(e)).

### 3.3. ceAF Promotes Wound Healing in Diabetic Mice

**Figure 3:**
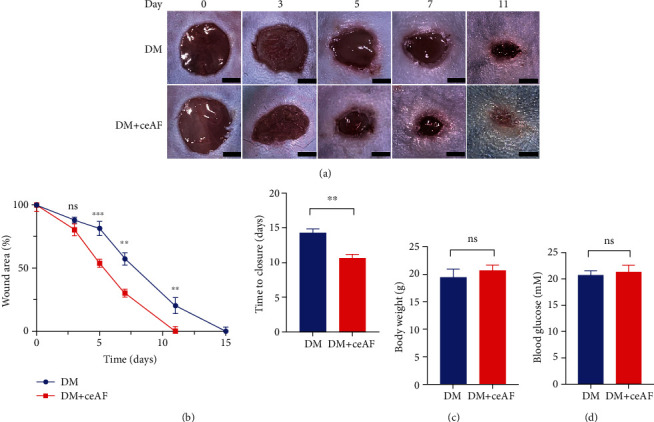
Treatment with ceAF ameliorates wound healing in STZ-induced diabetic mice. (a) Data for wound changes were recorded daily from day 1 to day 11. (b) Statistical graph representation of wound area and time to closure. (c) The measured body weight of the experimental mice on day 11. (d) The measured blood glucose of the experimental mice on day 11. Scale bar: 3 *μ*m. *n* = 6, ns: no statistical significance, ^∗∗^*P* < 0.01, ^∗∗∗^*P* < 0.001.

To evaluate the rates of wound healing in diabetes mellitus (DM) and treatment (DM+ceAF) groups, 8 mm full-thickness wounds were created on the back of the diabetic mice. As shown in Figures 3(a) and 3(b), the wound healing slowed down obviously in the DM group. The adverse trend was reversed in the DM+ceAF group from the fifth day (Figures 3(a) and 3(b)). To exclude confounding factors, we measured the body weight and blood glucose of the mice on day 11 and found no statistical difference between the two groups (Figures 3(c) and 3(d)).

### 3.4. ceAF Ameliorates the Wound Histological Indicators of Diabetic Mice

**Figure 4:**
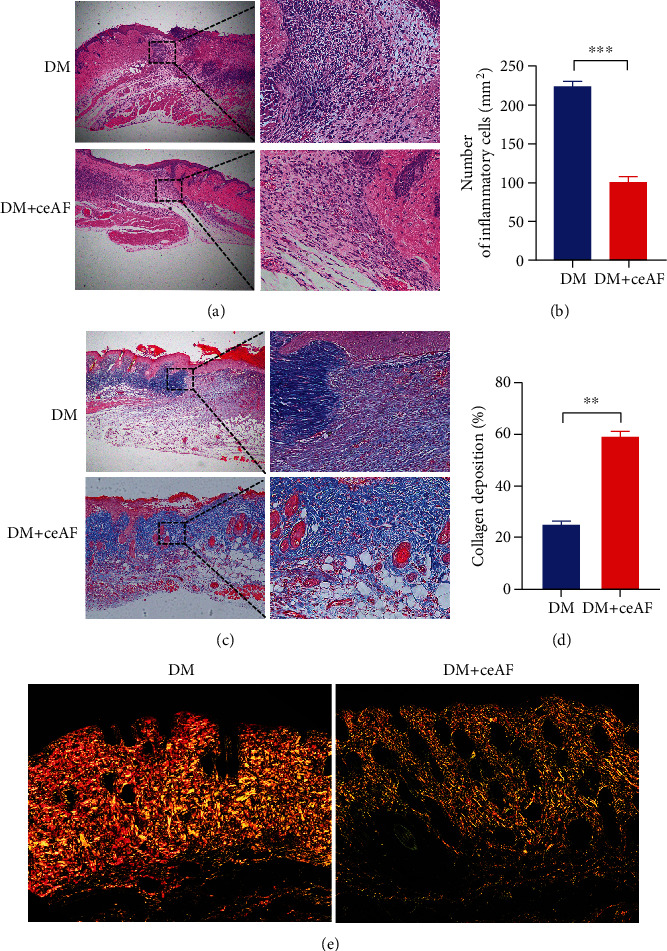
Effect of ceAF treatment on the histological changes in wound tissues. (a) H&E staining of skin wounds on day 5 indicated an enhanced inflammatory cell infiltration in diabetic mice treated with ceAF (Left 40×, Right 400×). (b) Histogram representation of inflammatory cell number. (c) MT staining of skin wounds on day 10 indicated an enhanced collagen deposition in the ceAF treatment group (Left 40×, Right 400×). (d) Histogram representation of collagen deposition. (e) Sirius red staining of DM group and ceAF+DM group after healing (40×). *n* = 6, ^∗∗^*P* < 0.01, ^∗∗∗^*P* < 0.001.

The protective effects of ceAF on diabetic wounds in mice were further evaluated by histopathological analysis. H&E staining analysis revealed an obvious difference in skin margin tissue between DM and DM+ceAF groups (Figure 4(a)). After ceAF administration, the inflammatory cells of diabetic mice wounds were markedly decreased compared to the control group (Figure 4(b)). Wound samples were collected on day 10 for the MT test. The results demonstrated that the proportion of collagen deposition was much higher in the DM+ceAF group than in the DM group (Figures 4(c) and 4(d)). The content of type I and III collagen in the wound after healing can reflect the healing quality to a certain degree. Therefore, we used Sirius red staining to determine the type of collagen in the newborn skin after wound healing. The staining results showed that the green coloration of type III collagen is more obvious in the DM+ceAF group. In contrast, the DM group contained more yellow-red type I collagen (Figure 4(e)).

### 3.5. ceAF Promotes M2 Macrophage Polarization and Neovascularization in the Wound of Diabetic Mice

**Figure 5:**
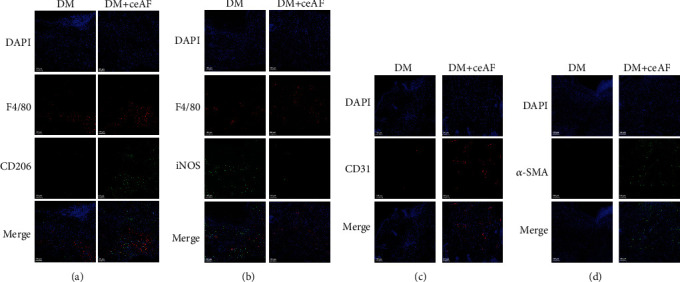
(a, b) Immunofluorescence assessment of CD206^+^ (green), iNOS^+^ (green), and F4/80^+^ (red) cells in DM and DM+ceAF on day 5 postinjury. (c, d) Immunofluorescence analysis of CD31^+^ (red) and *α*-SMA^+^ (green) cells in DM and DM+ceAF on day 10 postinjury. *n* = 6, Scale bar: 100 *μ*m.

Subsequently, we assessed the trend of macrophage polarization and angiogenesis *in vivo*. The immunofluorescence staining data indicated that the number of CD206^+^ macrophages was much higher in the DM+ceAF group than in the DM group on day 5 after wounding (Figure 5(a)). Meanwhile, the M1 macrophage population notably reduced in diabetic mice treated with ceAF (Figure 5(b)). The rate of wound vascularization is a crucial indicator for determining the effect of wound healing. Therefore, CD31 and *α*-SMA expression were further confirmed by immunofluorescence. It was observed that the DM+ceAF group had lesser CD31^+^ and *α*-SMA^+^ cells compared to the DM group on day 10 (Figures 5(c) and 5(d)).

### 3.6. ceAF Inhibits Inflammation through Inducing M2 Macrophage Polarization in the Wound of Diabetic Mice

**Figure 6:**
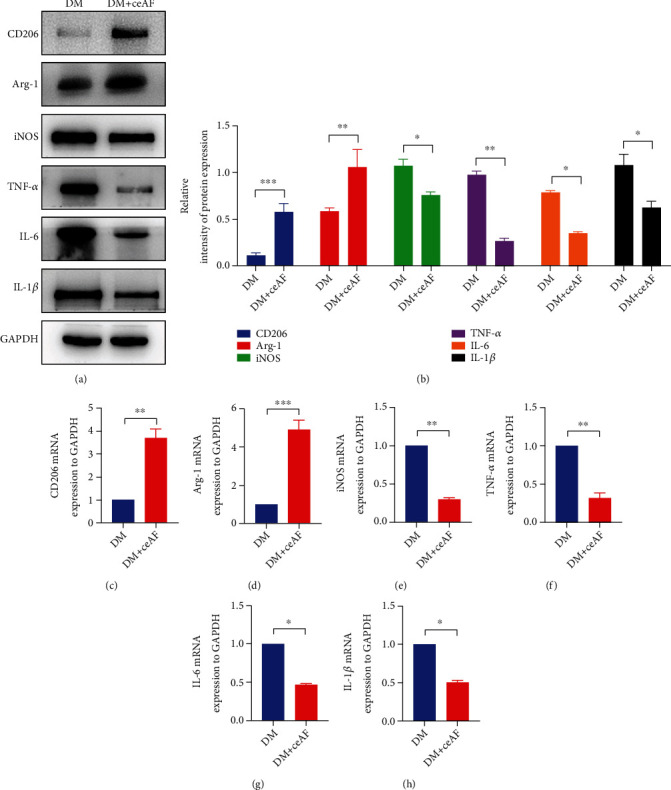
ceAF was able to suppress inflammation via promoting M2 macrophage polarization *in vivo*. (a) The expression of CD206, Arg-1, iNOS, IL-1*β*, IL-6, and TNF-*α* in the diabetic wound of mice was evaluated by WB. GAPDH served as a standard reference. (b) WB blots in (a) were quantified by densitometric analysis. (c–h) The expression of CD206, Arg-1, iNOS, IL-1*β*, IL-6, and TNF-*α* in the diabetic wound of mice was tested by RT-qPCR. *n* = 6, Mean ± SEM. ^∗^*P* < 0.05, ^∗∗^*P* < 0.01, ^∗∗∗^*P* < 0.001.

To verify the changes induced by ceAF therapy between macrophage polarization and inflammation *in vivo*, we collected mouse wound tissue on day 5 to verify the indicators of macrophages and inflammation using WB and RT-qPCR. The protein levels of CD206 and Arg-1 in the DM+ceAF group were remarkably elevated, and the expression of iNOS was dramatically weakened in comparison with that of the DM group (Figures 6(a) and 6(b)). The changes in proinflammatory factors were consistent with the M1 macrophage indicator. ceAF treatment markedly reduced the protein levels of TNF-*α*, IL-1*β*, and IL-6 compared to the DM group (Figures 6(a) and 6(b)). The transcriptional levels of the above indicators were measured by RT-qPCR. The experiments were repeated 3 times and similar trends were observed (Figures 6(c)–6(h)).

## 4. Discussion

A diabetic ulcer is a common complication in diabetic patients, and one of the reasons for a nonhealing wound is an excessive inflammatory reaction [21]. Herein, the anti-inflammatory effects of ceAF on diabetic wound healing and its underlying mechanism were elucidated. The main results are as follows: (i) ceAF could inhibit cellular inflammation by deactivating the TLR4/NF-*κ*B axis and activating the Nrf2 axis; (ii) ceAF can promote wound healing and improve histological indicators in diabetic mice; (iii) ceAF regulates the macrophage polarization both *in vivo* and *in vitro*; and (iv) ceAF ameliorates inflammation and oxidative stress by promoting M2 macrophage polarization. It is speculated ceAF exerts an anti-inflammatory function by promoting a rapid transition from the inflammatory stage to the remodeling stage. Our findings provide an important theoretical basis for treating refractory skin wounds in diabetes patients.

Amniotic fluid is essential for fetal development and survival, and its role varies at different stages of embryonic development [22]. The reason we chose early amniotic fluid is that it contains more growth-promoting substances such as stem cells, growth factors, and chemokines [23, 24]. Late amniotic fluid contains some urine and feces and may not be a good choice. It has been recently reported that amniotic fluids from different species have essential factors that can be used in a broad range of therapeutic areas such as corneal wound regeneration, diabetic wound care, and fetal wound healing [25, 26]. Nevertheless, the precise mechanisms remain unknown. Although we are still in the early stages of exploring the mechanism, the unique advantage of the current study is that we use eggs to separate amniotic fluid, which can be prepared on an industrial scale. The approach is economical, safe, and ethical, which further can be used for in-depth mechanism studies and preclinical trials.

Wound healing is mainly classified into four phases: hemostasis, inflammatory, proliferative, and remodeling [27]. All processes are intertwined, and persistent inflammation can have a range of negative effects on wound healing [28]. The evolution of a diabetic wound does not follow the normal healing time process and is affected by several factors such as hyperglycemia, chronic inflammation, microcirculation disorder, hypoxia, and autonomic neuropathy [29]. In diabetic wound, there are more inflammatory cell infiltration around the dermis and blood vessels, releasing a large number of reactive oxygen species and proteolytic enzymes, which continue to damage normal tissues [30]. In addition, the ratio of M1 (proinflammatory) to M2 (anti-inflammatory) macrophages has been in a heightened state [7]. The expression of inflammatory factors (e.g., TNF-*α*, IL-6, and IL-1) also continued to be high in diabetic wounds [6]. According to our results, ceAF treatment increased the ratio of M2 to M1 macrophages, thus suppressing inflammatory responses and promoting tissue repair.

It is believed that oxidative stress plays a critical role in diabetic wound healing. The imbalance of free radicals and antioxidants in patients leads to the overproduction of reactive oxygen species, which leads to cellular and tissue damage and delayed wound healing. ROS are key regulators of several stages of wound healing. In fact, low levels of ROS are necessary to combat external damage. However, excessive oxidative stress and decreased antioxidant capacity of tissues lead to redox imbalance, which is the main cause of diabetic wound nonhealing. Histological investigations have shown that nonhealing diabetic wounds are infiltrated by a highly oxidized environment, which is associated with hyperglycemia and tissue hypoxia, resulting in delayed wound repair [31]. Thus, reducing overproduction ROS levels and suppressing oxidative stress through the antioxidant system may reduce tissue damage and thus improve diabetic wound healing.

The polarization of macrophages is closely associated with wound healing. In normal wound healing, M1 macrophages dominate on days 1-3 and then transform into M2 macrophages [8]. The phenotype of macrophages in diabetic wounds is mostly M1 type, which will not decrease over time and rarely transforms into M2 macrophages [32]. There is an urgent need to suppress M1 polarization since the increased levels of M1 macrophages can exacerbate the progression of chronic ulcers [33]. There were several inflammation-associated factors present during diabetic wound healing, contributing to delayed wound healing during the inflammation phase. In chronic wounds, TNF-*α* and IL-6 increased, which led to elevated levels of metalloproteinases, degrading the extracellular matrix in the area and impairing cell migration [34, 35]. Flow cytometry and cell immunofluorescence showed that ceAF could induce RAW264.7 polarization to M2 type after 48 h. This preliminary evidence suggests that ceAF can inhibit inflammation by inducing polarization direction. *In vivo* experiments further confirmed this phenomenon, both the transcriptional and translational levels of M2-related genes in the wound tissue of ceAF-treated mice significantly elevated. Increasing M2 marker expression was accompanied by decreasing M1 marker and inflammatory cytokine expression, both of which occurred simultaneously. It proves partially that ceAF inhibits inflammation by improving the polarization of M2 macrophages.

In the canonical pathways, TLR4/NF-*κ*B and Nrf2 pathway activation is a fundamental step of inflammation launch [36]. TLR4 plays a pivotal role in the innate immune system and modulates it might offer therapeutic benefits for inflammatory diseases [37]. NF-*κ*B activation is responsible for the secretion of proinflammatory cytokines when TLR4 acts as a receptor for LPS [38]. As a result, NF-*κ*B has been reported as a potential target for treating inflammation. Furthermore, activation of the Nrf2 pathway could provide an endogenous defense system to resist cellular oxidative stress and mitigate oxidative damage, making it a dependable therapeutic method for suppressing wound inflammation. To explore the exact mechanisms underlying LPS-induced inflammation in RAW264.7 cells, we investigated whether ceAF affects TLR4 and NF-*κ*B activation. It was found that ceAF significantly decreased the LPS-stimulated upregulated expression of TLR4/NF-*κ*B pathway proteins, and the expression of related inflammatory factors was also inhibited. Combined with flow cytometry results, we speculate that ceAF switching RAW264.7 to M2 phenotype may contribute to TLR4/NF-*κ*B and Nrf2 axes.

In addition, macrophages polarized to M2 in diabetic wounds are beneficial to collagen production and angiogenesis [39]. M2 macrophages are responsible for the production of many proangiogenesis factors during wound healing, such as VEGF and EGF [40]. It was shown that macrophages can also induce fibroblast activation, as the paracrine factors from M1/M2 macrophage polarization provoked distinct fibroblast phenotypes [41]. Activation of fibroblasts forces extracellular matrix (ECM) formation which is closely related to collagen deposition [42]. Although excessive collagen deposition can lead to scar formation, the priority for diabetic wounds is to heal as soon as possible rather than scar treatment. The results of our study showed that ceAF-treated mice had increased angiogenesis on wound tissue and the main component of new collagen is type III. It suggests that ceAF can both improve the speed and quality of healing. This may be related to the fact that ceAF promotes macrophages to secrete more TGF-*β*1 and IL-10. More studies are needed to confirm the mechanism due to we only conducted relevant cell experiments.

## 5. Conclusion

In summary, our data suggest that ceAF can improve the inflammation and oxidative stress of LPS-stimulated RAW264.7 cells *in vitro* and promote STZ-induced diabetic wound healing *in vivo*. This function is achieved by regulating TLR4/NF-*κ*B and Nrf2 axis. Our findings provide a promising therapeutic strategy for treating diabetic wounds through the anti-inflammatory activity of ceAF.

## Figures and Tables

**Table 1:** Primer sequences used for RT-qPCR.

Gene	Forward	Reverse
*CD206*	GAGGGAAGCGAGAGATTATGGA	GCCTGATGCCAGGTTAAAGCA
*Arg-1*	TTGGGTGGATGCTCACACTG	GTACACGATGTCTTTGGCAGA
*iNOS*	ACATCGACCCGTCCACAGTAT	CAGAGGGGTAGGCTTGTCTC
*TNF-α*	CTGAACTTCGGGGTGATCGG	GGCTTGTCACTCGAATTTTGAGA
*IL-6*	CCAAGAGGTGAGTGCTTCCC	CTGTTGTTCAGACTCTCTCCCT
*IL-1β*	GAAATGCCACCTTTTGACAGTG	TGGATGCTCTCATCAGGACAG
*GAPDH*	CCAGTATGACTCCACTCACG	GACTCCACGACATACTCAGC

## Data Availability

The datasets used and analyzed are available from the corresponding author upon reasonable request.
